# Optimized intermediate layer formation and electroless plating methods to obtain supported dense Pd surfaces

**DOI:** 10.55730/1300-0527.3651

**Published:** 2023-12-07

**Authors:** Gamze GÜMÜŞLÜ GÜR, Berna TOPRAK

**Affiliations:** 1 Department of Chemical Engineering, Faculty of Chemical and Metallurgical Engineering, Istanbul Technical University, İstanbul, Turkiye; 2 Synthetic Fuels and Chemicals Technology Center, Istanbul Technical University, İstanbul, Turkiye

**Keywords:** Dense metallic membrane, Pd, electroless plating, intermediate layer, low temperature, hydrogen

## Abstract

Dense metallic membranes, especially Pd and Pd alloys, have been intensely investigated to provide an alternative and economical way to obtain H_2_ with ultrahigh purity. To overcome the high cost of Pd, composite membrane structures that comprise a thin layer of Pd are utilized. However, it is a challenge to obtain a thin, dense, and uniform Pd layer on the support materials. This study investigates the parametric analysis of γ-Al_2_O_3_ interlayer formation and the electroless Pd plating (Pd ELP) procedures on α-Al_2_O_3_ supports with the aim to achieve a thin, uniform Pd surface without annealing. Adjustments in PEG/PVA concentration, dipping time, and heat treatment enabled creating a thin γ-Al_2_O_3_ interlayer on α-Al_2_O_3_, minimizing pore size and density. Hydrazine concentration, heat treatment, and bath temperature were adjusted to optimize Pd ELP to achieve maximum yield from the plating bath and a dense, uniform surface without annealing. Pd/γ-Al_2_O_3_/α-Al_2_O_3_ structures were analyzed using scanning electron microscopy, X-ray diffraction, and thermogravimetric analysis to observe the impact of varied parameters on surface structures. Optimized sample was compared to an annealed Pd/α-Al_2_O_3_ prepared in accordance with literature methods and a Pd/graphite/α-Al_2_O_3_ sample to validate the use of optimized ELP procedure and the γ-Al_2_O_3_ interlayer. Results show that a dense and uniform 13 μm Pd coating was achieved on a γ-Al_2_O_3_-coated α-Al_2_O_3_ support without annealing, using three fresh ELP baths. This was done using sequential hydrazine addition with a decreased concentration (1 M) into the ELP baths at 30 °C, and applying heat treatment at 120 °C between each fresh ELP bath.

## 1. Introduction

Hydrogen plays a pivotal role in the greenization, decarbonization, and transition to renewable energy production [1–5]. It can be derived from various sources, including renewable ones like biomass, solar energy, water, and wind power, as well as nonrenewable sources such as petrochemicals. However, due to its nature, hydrogen typically occurs mixed with other gases, making the separation of hydrogen from these gas mixtures, whether from waste gases or synthesis gas (CO + H_2_), crucial for achieving long-term sustainability.

**Figure 1:**
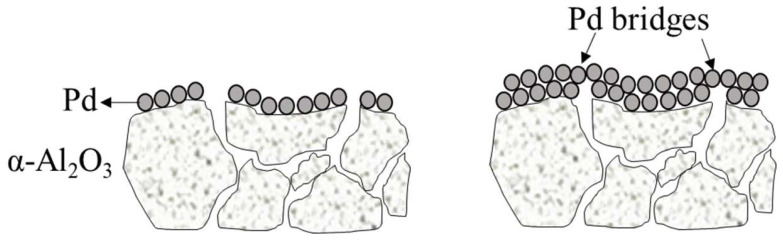
Schematic description of the bridge mechanism during ELP over the porous structure of α-Al_2_O_3_.

Membrane technology offers a viable solution to the challenge of H_2_ separation owing to its simplicity and cost-effectiveness compared to the conventional processes like pressure swing adsorption and cryogenic distillation [6–9]. Among the various options, dense Pd-based metal membranes have gained significant attention for hydrogen separation/purification. Separation of H_2_ from dense metallic membranes occurs in three steps: 1 - dissociative adsorption of H_2_ on the upstream side of the membrane, 2 - diffusion of H atom through the interstitial space within the bulk metal, 3 - recombinative desorption of H_2_ from the downstream side of the membrane. Considering this process as a whole reveals Pd as the prime candidate for membrane material due to its high H_2_ activity and near-infinite permselectivity [2,10–12]. However, the cost of Pd poses a challenge, prompting researchers to focus on fabricating thin palladium layers (1–15 μm) on support materials/substrates, which increases the mechanical stability of the membrane [13]. The choice of support material is crucial to achieve a thin and defect-free Pd layer, and α-Al_2_O_3_ has been extensively used as a support material. Al_2_O_3_ stands out as an ideal material for dense Pd membranes due to its excellent thermal stability and chemical inertness. which are essential for the high-temperature operations in H_2_ separation. Additionally, α-Al_2_O_3_ offers strong mechanical strength and hardness, which are crucial for maintaining membrane integrity. Moreover, thermal expansion coefficient of Al_2_O_3_ closely aligns with that of Pd, minimizing stress and risk of failure during the process. Economically, it is cost-effective and widely available, making it suitable for large-scale applications. Suitability of Al_2_O_3_ for H_2_ separation processes can be further confirmed by its proven track record in catalysis and separation technologies, along with the ability to be shaped into various forms which offers flexible design opportunities [10,11,13–18]. Nonetheless, α-Al_2_O_3_ substrates typically have large pores (5–200 nm), which hinders the formation of a dense Pd layer. As illustrated in Figure 1, during Pd deposition, Pd atoms aggregate form bridges over the pores by the bridge mechanism [19]. However, large pores obstruct the formation of these Pd bridges, resulting in surface defects. To overcome this limitation, an intermediate layer can be introduced to reduce the pore size and ensure a narrow pore size distribution on the alumina surface [13,17,20]. Among the various intermediate layers explored, γ-Al_2_O_3_ has been frequently utilized [12,21–23].

Several studies have reported successful fabrication of palladium membranes on modified α-Al_2_O_3_ supports. For instance, Zhao et al. [24] prepared a 1-μm thick and thermally stable Pd membrane by modifying the substrate with a Pd(II)-modified boehmite sol using the sol-gel process. Similarly, Zhang et al. [25] obtained a thin, defect-free Pd composite membrane on a γ-Al_2_O_3_-modified substrate. Pan et al. [21] deposited an ultrathin Pd layer (<3-μm-thick) on a Pd/γ-Al_2_O_3_ layer (<4-μm-thick) coated hollow α-Al_2_O_3_ support. Tanaka et al. [26] fabricated a composite membrane packed with Pd nanoparticles under vacuum on a mesoporous yttria-stabilized zirconia and γ-Al_2_O_3_ intermediate layer. Nair et al. [27] reported a reduction of approximately 60% in Pd membrane thickness with an increased H_2_ flux using a γ-Al_2_O_3_ intermediate layer. Lim and Oyama [28] prepared a defect-free Pd-Cu membrane, with a considerable amount of H_2_ permeance, on a porous alumina substrate coated with γ-Al_2_O_3_ intermediate layer with a pore size of 5 nm. The studies in the literature utilize boehmite sols and dipping solutions that comprise either or both of polyvinyl alcohol (PVA) and polyethylene glycol (PEG) with different concentrations. PVA and/or PEG are used in application to avoid defects, such as cracks, on the γ-Al_2_O_3_ surface. However, the literature lacks a systematic understanding of how the dipping time and PVA/PEG concentrations of the sol-gel process affect the γ-Al_2_O_3_ and plated Pd surfaces.

Various techniques can be employed for Pd deposition, such as physical/chemical vapor deposition, electrochemical deposition, and electroless plating (ELP) [10,13]. Among these techniques, ELP is the most effective, long-lasting, and simple method that enables effective deposition of the Pd layer regardless of the surface structure or conductivity [29]. Prior to ELP, a seeding step is generally required to create the anchor sites on the surface for plating to occur [13]. Additionally, an annealing step, often lasting long hours, even days, is typically performed after ELP to provide a uniform surface [30–32]. Consequently, the seeding, plating and annealing procedures individually play crucial roles in achieving a uniform and dense Pd layer. Traditional methods utilize SnCl_2_-containing baths for the seeding step [33], and EDTA-containing baths are used for plating [29,34], but these techniques lead to Sn and carbon contamination of the Pd layer, respectively [16,35,36]. Paglieri et al. [26] introduced an alternative seeding method that eliminates the need of SnCl_2_, and Thoen et al. [37] presented a plating method that excludes the use of EDTA. Another crucial aspect of the ELP process is the use of hydrazine as a reducing agent. Hydrazine is generally listed in the reducing solution recipe to activate the surface after seeding process. However, it plays a primary role in the plating step as one of the reactants. Literature studies suggest that the final film structure is determined by the plating rate which depends on various factors, including reactant concentration [31,38,39]. Hydrazine, a reactant, can induce bath decomposition or decreased mass transfer if not used in the correct amount. This can result in nonhomogeneous surface structures that cannot be utilized as membranes [27]. The ambiguity in the literature regarding the concentration and use of hydrazine in the plating step remains a problem in achieving the formation and reproducibility of a homogeneous Pd surface.

Although defect-free Pd surfaces can be achieved with successive plating baths and long annealing steps, it is important to note that the current plating procedures still result in a significant loss of Pd in the process, especially due to the lack of information in hydrazine use. In other words, the efficiency of Pd transfer from the solution to the support surface is low. Additionally, the days-long annealing process not only prolongs the overall membrane preparation time but also leads to unnecessary energy losses. Therefore, in this study, the aim is to explore and optimize the methods and process parameters to maximize the Pd transfer efficiency in ELP and minimize the membrane preparation time. We first optimized the sol-gel process to modify the support surface with γ-Al_2_O_3_ to decrease the density and the size of the pores to achieve a thin, defect-free Pd surface. Optimization of the sol-gel process involved adjusting sol-gel concentration, application time, volumetric addition percentage of PVA and PEG, and calcination procedure. Next, we conducted a parametric study to investigate the effects of plating duration, bath temperature, hydrazine concentration and introduction methods, as well as interstage drying. The optimized procedure yielded a thin, dense, defect-free Pd layer without the need for the annealing step.

## 2. Materials and methods

### 2.1. Materials

Alpha aluminum oxide (α-Al_2_O_3_) with a purity of 99.9% in powder form was purchased from Alfa Aeser Brockmann. Palladium (II) acetate (C_4_H_6_O_4_Pd) with a purity of 99.9% and Palladium (II) chloride (PdCl_2_) with a purity of 99.9% were obtained from Aldrich Chemistry. Chloroform with a purity of 99.9%, ammonium solution (NH_4_OH) with a purity of 25% ammonium solution (NH_4_OH), 35% hydrogen peroxide (H_2_O_2_), 37% hydrochloric acid (HCl), 65% nitric acid (HNO_3_) are purchased from ISOLAB. Hydrazine (N_2_H_4_) 65% hydrazine (H_4_N_2_) and PEG (HO(C_2_H_4_O)_n_H) are purchased from Sigma-Aldrich. Lastly, PVA (C_2_H_4_O)_n_ is obtained from abcr GmbH. Boehmite powder was obtained from ETI-Maden A.S.

### 2.2. Preparation of α-Al_2_O_3_ membrane supports

The α-Al_2_O_3_ supports were prepared by pressing α-Al_2_O_3_ powder (with particle size less than 100 μm) into tablets (7 mm dia., 3 mm thick) using an isostatic press under 400 MPa. The tablets were then tablets were calcined under H_2_ atmosphere at 600 °C. After calcination, α-Al_2_O_3_ tablets were heated under Argon gas (Ar) to 300 °C with a 2.5 °C/min heating rate. Once the temperature reached 300 °C, H_2_ was introduced into the system, and the temperature was further increased to 600 °C at the same heating rate. The tablets were kept at 600 °C for 2 h in a mixture of H_2_ and Ar. Following calcination, the tablets were cleaned in acetone using an ultrasonic bath to remove all possible oil-based dirt that may have adhered to the surface during handling. Subsequently, the tablets were dried at 70 °C for 3 h and weighed.

### 2.3. Preparation and application of γ-Al_2_O_3_ coating

The sol synthesis formulation followed the procedure described by Zhang et al. [25]. Boehmite powder was slurried with DI water and stirred at 200 rpm and 90 °C for 1 h to obtain a 0.5 M boehmite sol. To peptize the boehmite powder, 1.6 M HNO_3_ was added to the mixture. Subsequently, the solution was refluxed with continuous stirring at 80 °C. After 5 h of refluxing, 1.2 (wt.) % PVA and 0.6 (wt.) % PEG (PVA:PEG = 2:1) were added to the refluxed boehmite sol, resulting in an AlOOH dip-coating solution. α-Al_2_O_3_ supports were then dipped into prepared boehmite-sol for 30 s. Finally, γ-coated supports-coated supports were dried at room temperature for 24 h and further heated to 600 °C at a heating rate of 1 °C/min. The tablets were calcined at 600 °C for 3 h, resulting in the transformation of the AlOOH structure to γ-Al_2_O_3_ phase. The sol concentration, coating time (in seconds), volumetric percentage of PVA and PEG, and the calcination procedure were optimized to achieve a uniform, thin γ-Al_2_O_3_ intermediate layer on the α-Al_2_O_3_ surface.

### 2.4. Preparation of Pd surfaces

The Pd surface preparation procedure consists of two steps: 1 - activation of membrane surface with Pd nuclei, identified as seeding, and 2 - formation of the Pd layer on the activated surface through electroless plating. To ensure that Pd plating occurred only on one surface of the tablets, the side and the other face of the tablets were sealed with Teflon tape. Activation of the support surface was performed using the procedure described by Paglieri et al. [16]. To form Pd nuclei on the surface, tablets were dipped into the seeding solution composed of Pd-acetate and chloroform for 3 min. Subsequently, the tablets were immersed in a H_2_O_2_ solution (3 wt. %) for 30 min at room temperature to decompose the acetate group. To obtain Pd nuclei, the tablets were then placed into a reducing solution and kept at 50 °C for 20 min while being vigorously shaken. The contents and compositions of each solution are summarized in Table 1.

For Pd plating, the EDTA-free procedure described by Gade et al. [36] was employed with additional modifications. In this procedure, the plating solution was initially prepared (details given in Table 1) without the hydrazine. This solution was poured in a vial containing the Teflon covered tablet and placed into a shaking water bath (200 rpm) at 50 °C. Once the solution reached 50 °C, hydrazine was injected into the solution which immediately initiated Pd plating. The plating solution was changed every hour to achieve a uniform Pd coating. Upon completion of the ELP, the tablets were soaked into DI water at 70 °C and then dried at 120 °C overnight.

### 2.5. Characterization of the samples

α-Al_2_O_3_, γ-Al_2_O_3_, and Pd surface features and crystallinity were analyzed by scanning electron microscopy (SEM- JEOL JSM-6390LV) and X-ray diffraction (XRD-Panalaytical Aeris model). The Pd layer thickness was estimated using the gravimetric method. Initial weight was measured after the γ-Al_2_O_3_ layer was applied and the tablet was cleaned and dried. The final weight was measured following the drying of the cleaned Pd-plated sample. The difference between the initial and final weights was used to calculate the Pd thickness using Pd density and the surface area of the sample. Thermal behavior of calcined boehmite powder was analyzed with thermogravimetric analysis (TA Instruments TGA 55), and the weight loss of boehmite during the transformation to the γ-Al_2_O_3_ was observed.

## 3. Results and discussions

### 3.1. Pd coating on α-Al_2_O_3_ support – benchmark sample

**Figure 2:**
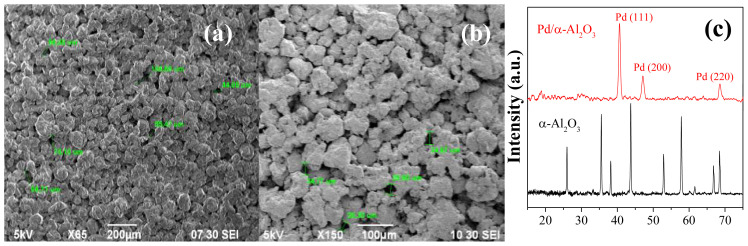
SEM images of an α-Al_2_O_3_ tablet (a) before and (b) after Pd ELP procedure and (c) the related XRD patterns.

The α-Al_2_O_3_ tablets that were prepared and cleaned as described above were initially used to create a benchmark sample for comparing the effectiveness of the optimized Pd surface preparation method. To analyze the surface structure, an SEM picture of a clean α-Al_2_O_3_ tablet (Figure 2a) was taken. The image reveals a rough surface with numerous wells and pores ranging in widths from 45 μm to over 100 μm. When the ELP was directly applied to this surface, a rough Pd coating was obtained, seen in Figure 2b, following the procedure described in the literature, which is plating for 3 h with fresh ELP bath every hour. The SEM image in Figure 2b suggests that it is difficult for Pd bridges to form on a rough surface with large holes, as it is in this case, leading to a nonhomogenous and noncontinuous Pd surface.

The presence of the Pd layer on this sample was confirmed by XRD analysis. The XRD pattern of the sample after ELP procedure exhibits significant Pd peaks with no contribution from the α-Al_2_O_3_ tablet, as shown in Figure 2c. The absence of α-Al_2_O_3_ peaks confirms the complete coverage of the surface with a thick layer of Pd. However, the state of the surface would most probably lead to nonselective operation if it was used as-is in the H_2_ separation process.

### 3.2. Preparation of γ-Al_2_O_3_ layer and optimization of procedure

**Figure 3:**
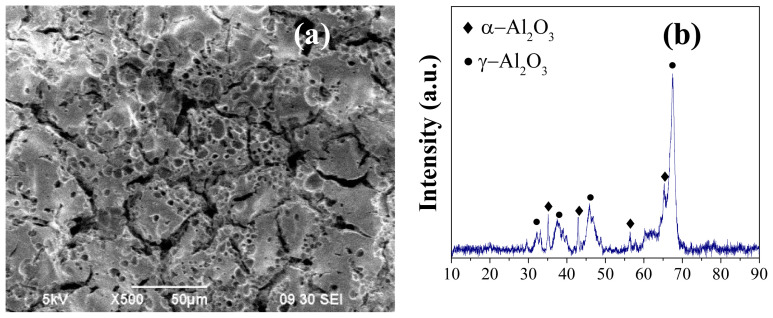
SEM image (a) and the XRD pattern (b) of the sample obtained with the initial γ-Al_2_O_3_ preparation procedure.

To decrease the surface roughness and pore size on the α-Al_2_O_3_ surface, an intermediate γ-Al_2_O_3_ layer was coated. Initially, the method introduced by Zhang et al. [25], as described earlier, was employed for this purpose. However, due to insufficient information in the literature, the concentrations of PVA and PEG for peptization were estimated. The initial concentrations of PVA and PEG in the solution were set at 2 and 1 (vol.) %, respectively, with a PVA/PEG ratio of 2. The literature also lacked precise details on the application time and calcination procedure. Thus, an initial application time of 10 s and direct heating to 600 °C, at which the γ phase formation occurs, at a heating rate of 3 °C/min, were chosen. The SEM image and the XRD patterns of the first sample are presented in Figure 3.

**Figure 4:**
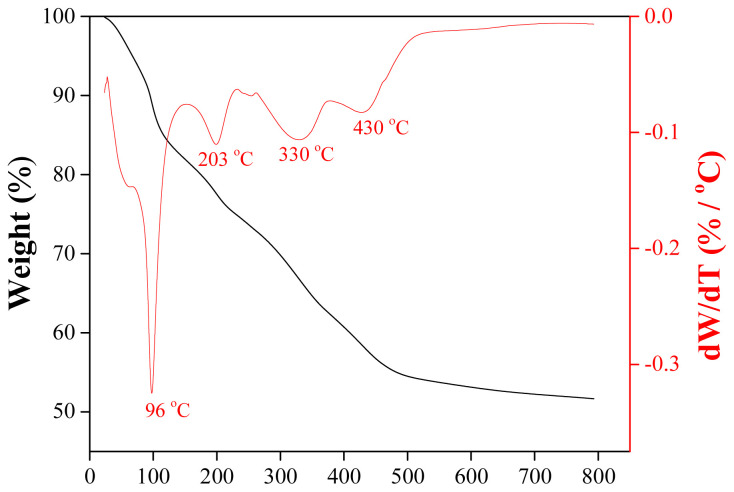
TGA and DTA profiles of boehmite powder.

Figure 3a shows a cheese-like structure of the γ-Al_2_O_3_ layer with large cracks across the surface. The XRD pattern (Figure 3b) confirms the formation of the γ-Al_2_O_3_ phase, the intended structure. Moreover, the presence of α-Al_2_O_3_ peaks suggests that γ-Al_2_O_3_ layer was coated as thinly as possible, as desired. Since 600 °C is not sufficient for the formation of α-Al_2_O_3_ phase, these signals originate from the tablet surface, indicating the thin nature of γ phase. To confirm this, TGA-DTA analysis of the boehmite powder was performed. As shown in Figure 4, the mass of the boehmite powder decreases drastically until 500 °C, with only a slight change thereafter. The first mass loss region (T < 100 °C) in the TGA profile is attributed to the desorption of physisorbed water, the second region (100–480 °C) to the transition into the γ-Al_2_O_3_, and the third region (T > 480 °C) to the removal of residual hydroxyls [40,41]. These mass losses are in agreement with the DTA curve which shows peaks at 96 °C, 203 °C, 330 °C, and 430 °C, corresponding to the evaporation of physically adsorbed water, decomposition of PVA and PEG, desorption of interstitial water trapped in boehmite (dehydration) and transition into γ-Al_2_O_3_ phase, respectively [42,43]. After 500 °C, no significant mass loss was observed until 800 °C. These results demonstrate that 600 °C is sufficient for the formation of γ-Al_2_O_3_ phase and does not lead to α-Al_2_O_3_.

**Figure 5:**
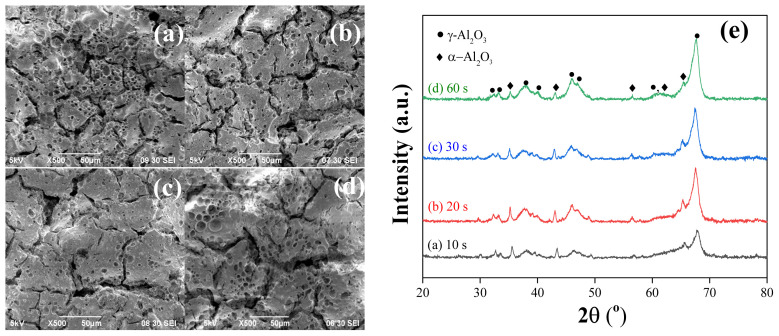
SEM images of the samples after (a) 10 s, (b) 20 s, (c) 30 s, and (d) 60 s of sol application and (e) the related XRD patterns.

The secondary layer application considerably reduced the surface roughness and porosity, but the presence of cracks and the cheese-like structure could impact the homogeneity of the Pd plating. Therefore, a series of modifications were performed on the application time, calcination procedure and PVA/PEG ratio, and their effects were observed. These modifications are summarized in Table 2 and the flowchart in Figure 5.

**Figure 6:**
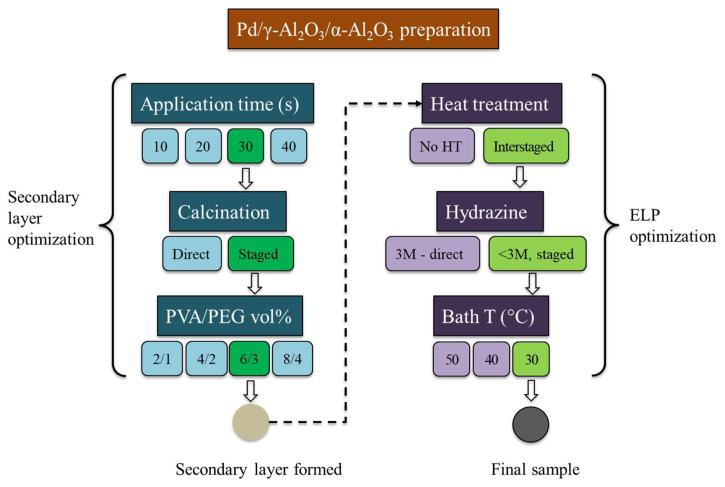
The flowchart summarizes the parametric analyses in this study and demonstrates the flow of experiments to optimize secondary layer formation and ELP procedures to achieve the final sample, Pd/ γ-Al_2_O_3_/α-Al_2_O_3_ membrane.

Increasing the application time from 10 to 30 s improved the surface structure, as can be observed in the SEM images in Figure 6a–6d. The increase in application time resulted in a decrease in the cheese-like structure formation and slightly reduced crack formation. However, further increase in application time did not improve the surface structure. On the contrary, the cheese-like structures and deeper cracks started to form in addition to significant thickness differences across the surface. The difference between the obtained surfaces can also be observed in the XRD patterns in Figure 6e.

The XRD patterns in Figure 6e show that the intensities of the γ-Al_2_O_3_ phase peaks increased with increasing application time. However, after 60 s of application, the α-Al_2_O_3_ phase peaks become smaller, indicating an increase in the thickness of the γ-Al_2_O_3_ layer. Therefore, the optimum application time was determined to be 30 s based on both the XRD and SEM analyses.

**Figure 7:**
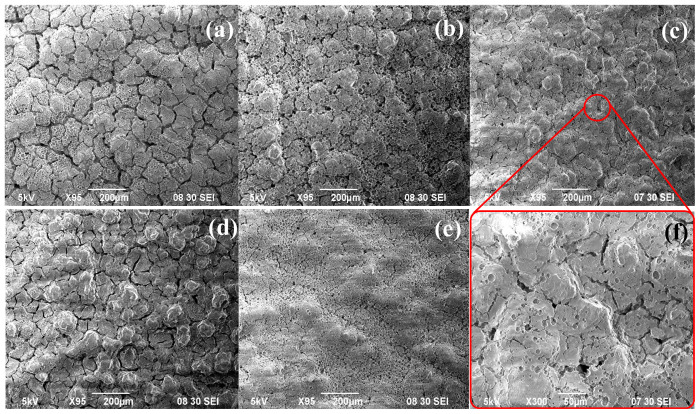
SEM images of surface coated using PVA/PEG concentration of (a) 4 / 2 (vol.) %, (b) 4 / 2 (vol.) % with staged heating, (c) 6 / 3 (vol.) %, (d) 8 / 4 (vol.) % (e) 6 / 3 (vol.) % with double dipping, and (f) zoomed in image (c).

As a next step, the effects of the PVA-PEG concentrations and the calcination procedure on the surface structure were examined. For this, PVA and PEG concentrations were doubled, tripled, and quadrupled, and the calcination method was changed from direct heating method to staged method. The surfaces obtained with varying PVA-PEG concentrations and calcination procedures were analyzed by SEM (Figure 7).

In the staged calcination, the sample was heated at a slower heating rate to an intermediate temperature of 300 °C. The sample was kept at that 300 °C for 30 min before heated to 600 °C, again at a heating rate of 1 °C/min. During cooling, the sample was first cooled to 300 °C and then to room temperature. This staged heating–cooling cycle aimed to provide longer time for the surface temperature to equilibrate with the ambient temperature, reducing strain. As seen in Figures 7a and 7b, the formation of cracks was decreased as expected. Consequently, the staged calcination procedure was employed in further experiments.

Figures 6c and 7b–7d demonstrate that the surface properties are affected by the PVA-PEG concentration in the sol. With increasing concentration, there was a significant reduction in the crack formation and the cheese-like structure, resulting in an improved surface state. However, a higher concentration of PVA-PEG (Figure 7d) led to more crack formation on the surface. To further analyze the surface properties, sample 7, previously dipped in the sol with PVA/PEG concentration of 6/3 (vol.) %, was dipped in the same sol a second time. This method, as seen in Figure 7e, resulted in a more homogeneous surface. Nevertheless, as seen from the zoomed in image of sample 7 in Figure 7f, the surface of sample 7 already exhibited acceptable structure for ELP application. Therefore, it would be unnecessary to spend additional time and materials to slightly improve the surface and increase the thickness of γ-Al_2_O_3_ layer. The experimental analysis revealed that using a sol with a PVA/PEG concentration of 6/3 (vol.) %, dipping the sample for 30 s into this sol, and applying staged calcination procedure enabled formation of a thin, homogeneous γ-Al_2_O_3_ layer on the α-Al_2_O_3_ surface.

### 3.3. Optimization of ELP procedure

The ELP procedures described in the literature generally oversimplify the plating procedure, lacking details of the application method or the number of baths to achieve a uniform, dense Pd layer. Therefore, a series of different samples were prepared with adjustments made to different parameters to optimize the ELP procedure and eliminate the need for annealing. The samples and preparation methods are summarized in Table 3 and Figure 5.

**Figure 8:**
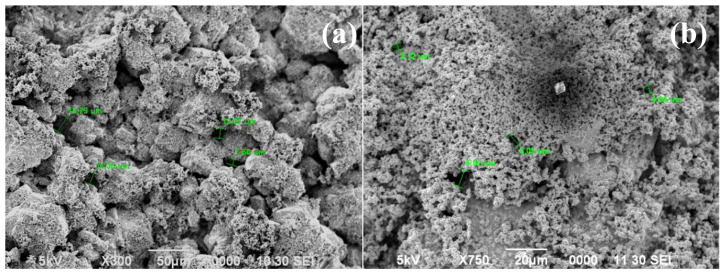
SEM images of the samples with (a) 7 consecutive ELP applications (sample 8), and (b) interstage drying after 3 ELP application followed by 4 more (sample 9).

Due to the ambiguity in the ELP procedure, as described in Section 3.1, the initial Pd ELP trial resulted in a nonuniform and noncontinuous Pd surface. To understand the effect of number of ELP baths, a new trial was conducted with a 7-h experiment, increasing the number of baths from 3 to 7. The surface structure after 7 baths can be observed in Figure 8a. Increasing the number of baths from 3 (Figure 2b) to 7 (Figure 8a) resulted in a different surface structure, but without significant improvement. Staged calcination procedure in the γ-Al_2_O_3_ layer coating proved to improve the surface state. Therefore, a similar procedure was applied in Pd layer formation. After using 3 fresh plating baths successively, the sample was cleaned in DI water and dried at 120 °C. Subsequently, the sample was again subjected to ELP application 4 more times refreshing baths every hour. The SEM image of the sample prepared using 3 + 4 ELP baths with interstage drying (Figure 8b) shows an improved Pd surface compared to the sample prepared by 7 consecutive ELP applications. It is known that heat treatment is effective in determining the crystallinity and the crystal structure of metals [44,45]. It can be concluded that a heating step between the ELP applications allows the surface to relax, resulting in a more homogeneous deposition of Pd over the surface. Therefore, the ELP procedure was modified to include interstage drying between each ELP bath. Another interesting finding in the SEM image of the 3 + 4 ELP sample is the presence of a square shaped particle on the surface, believed to be a piece of Pd metal that precipitated in the solution. This phenomenon requires additional investigation, which will be discussed below.

**Figure 9:**
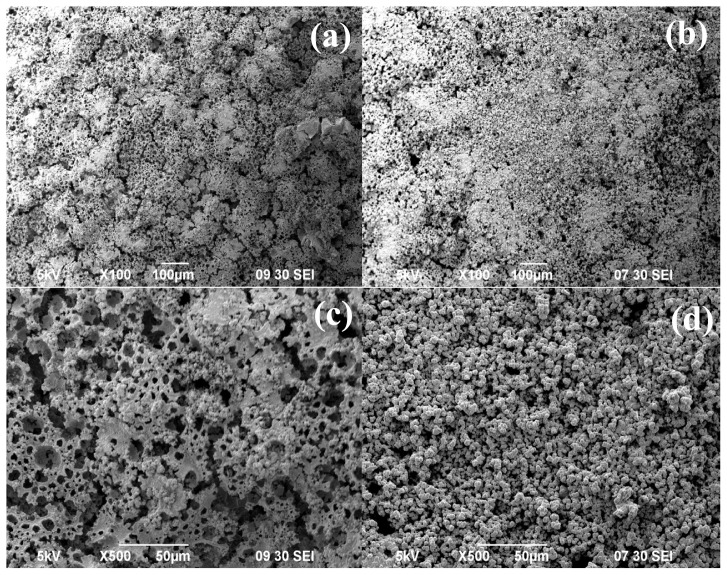
SEM images of samples after 3 ELP applications prepared adding (a) 3 M hydrazine at once (sample 10) and (b) 1 M hydrazine three times sequentially (sample 11) with interstage heating between each bath at 100× (a, b) and at 500× (c,d) magnification.

The modified ELP procedure was applied to the previously prepared sample with the γ-Al_2_O_3_ layer (sample 10). This sample was subjected to 3 ELP applications with interstage drying between each application. The SEM images of the surface shown in Figures 9a and 9c demonstrate that even with only 3 ELP applications, an improved Pd surface can be achieved if interstage heating is employed. However, the sponge-like structure and the presence of Pd precipitates in the bath and on the surface, as shown in Figure 8b, still remained as issues to be solved.

The deposition of Pd in ELP occurs through the chemical reaction given below:


(1) $$2Pd^{+2}+N_{2}H_{4}+4\;OH^{-}\to 2Pd+N_{2}+4H_{2}O$$

Equation (1) represents the most generic form of the Pd ELP reaction that can be found in the literature. Hydrazine (N_2_H_4_), as the reducing agent, is shown as one of the reactants of this reaction. However, its significance in the reaction kinetics is higher than it is credited for in the literature. During the ELP applications, it was observed that the color of the bath instantly changed from clear to black upon addition of 3 M hydrazine at a 1 (vol.) % amount into the solution at 50 °C. This rapid color change was also accompanied by bulk Pd precipitation. These facts indicate that the bath solution, already unstable by the absence of EDTA, decomposes due to the high bath temperature and/or high hydrazine concentration [27, 38, 39, 46]. This leads to Pd precipitating in the solution rather than attaching to the tablet surface, reducing the deposition yield. Therefore, the hydrazine concentration and bath temperature were modified, and their effects on ELP efficiency were investigated.

In the following experiment, the concentration of hydrazine was decreased from 3 M to 1 M. However, to effectively utilize the amount of Pd^+2^ ions in the bath, a new method inspired by the work of Wang et al. [47] was applied. In that study, after the regular concentrated hydrazine addition to the bath, rather than changing the bath, more hydrazine was added to the same bath. This leads to increased hydrazine concentration in the bath and does not alter the initial plating rate. In our study, instead of refreshing baths or increasing hydrazine concentration by successive addition as done by Wang et al. [47], after the initial 1 M, an additional 1 M of hydrazine was introduced to the bath every hour for 2 h. For each new bath, the hydrazine addition was performed in three steps, each with equal volumes of a 1 M solution. This way, the total number of moles of hydrazine added to the solution was kept the same as before, but the addition was performed with a decreased hydrazine concentration, resulting in a decreased rate of reaction. The SEM images of sample 11 given in Figures 9b and 9d show that the sequential addition of hydrazine with decreased concentration results in a more uniform and continuous Pd surface. However, this surface, without any additional treatment, is still not acceptable as a membrane surface.

**Figure 10:**
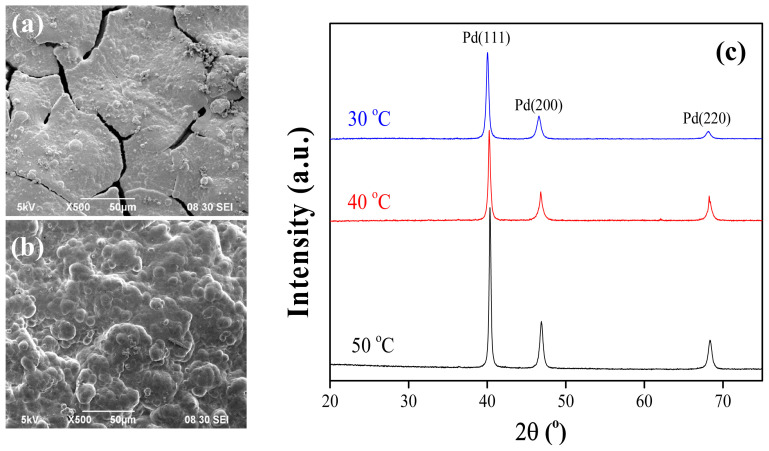
SEM image of the samples prepared using ELP baths at (a) 40 °C, (b) 30 °C, and (c) the XRD patterns of the samples prepared at 50 °C, 40 °C, and 30 °C.

It was observed that slowing the reaction rate through controlled hydrazine introduction leads to a more homogenous surface. This suggests that slowing the plating rate even further could improve the surface. Decreasing the hydrazine concentration or amount further is not feasible as it is one of the reactants in the reaction. Therefore, an attempt was made to slow down the reaction by decreasing the bath temperature. Two separate experiments were performed with bath temperatures set as 40 and 30 °C. The SEM images of these samples are presented in Figure 10.

The SEM images given in Figures 10a and 10b represents how the final surface is affected by the bath temperature. Decreasing the temperature to 40 °C led to the formation of more uniform structures separated by significant cracks. Further decreasing the bath temperature to 30 °C enabled formation of a homogeneous and continuous Pd surface. This suggests that the decrease in temperature resulted in slower reaction kinetics, allowing enough time for Pd ions to reach and attach to the surface rather than precipitating in the bulk solution. The XRD patterns of these three samples are almost the same with a slight difference in the intensity of the peaks, as seen in Figure 10c. This is an indicator of the change in the crystallinity. The average crystallite sizes on the surfaces were calculated using Scherrer equation with the first two significant peaks in the XRD patterns and listed in Table 3. These values demonstrate that the crystallite size on the surface decreases considerably by decreasing the bath temperature from 50 to 40 °C. However, the crystallite sizes estimated at 40 and 30 °C are similar, demonstrating that further decrease in bath temperature does not affect the crystallite size significantly. The crystallite size, also referred to as grain size in the literature, is an important feature that affects membrane performance as it is a characteristic measure of the microstructure [48]. Studies in the literature claim that the permselectivity of the membrane increases with decreasing crystallite (grain) size [27,48]. Larger grains lead to larger grain boundary gaps that decrease the permselectivity by allowing all molecules to pass through. Additionally, larger grain sizes make the material brittle, thus the membrane becomes more prone to deformation [49–51]. Therefore, it can be expected to achieve a better stability and H_2_ permselectivity with surfaces prepared using baths at 40 and 30 °C, disregarding the surface defects/cracks on sample prepared at 40 °C. It can be concluded that the Pd surface obtained in this study at 30 °C is more suitable for achieving better stability and H_2_ permselectivity, as it possesses a small grain size as well as a dense, homogenous Pd surface.

In summary, it can be concluded that there are two factors at play during ELP: reaction rate and mass transfer rate. Based on the experiments conducted, it was concluded that the mass transfer is slower than the reaction itself at high temperatures and high hydrazine concentrations, making the process mass transfer limited. This leads to the precipitation of Pd in the bulk solution before reaching the surface, due to high Pd ion concentrations in the solution. In addition, it results in larger grains on the surface that could cause embrittlement and nonselective transport. On the other hand, controlled addition of hydrazine with lower concentrations and lower bath temperatures slows down the rate of reaction and increases the chances for Pd ions to reach the surface. The key to the successful plating is to find the optimum conditions at which reaction rate is slowed such that it becomes the limiting rate of the plating process.

### 3.4. Comparison of methods

**Figure 11:**
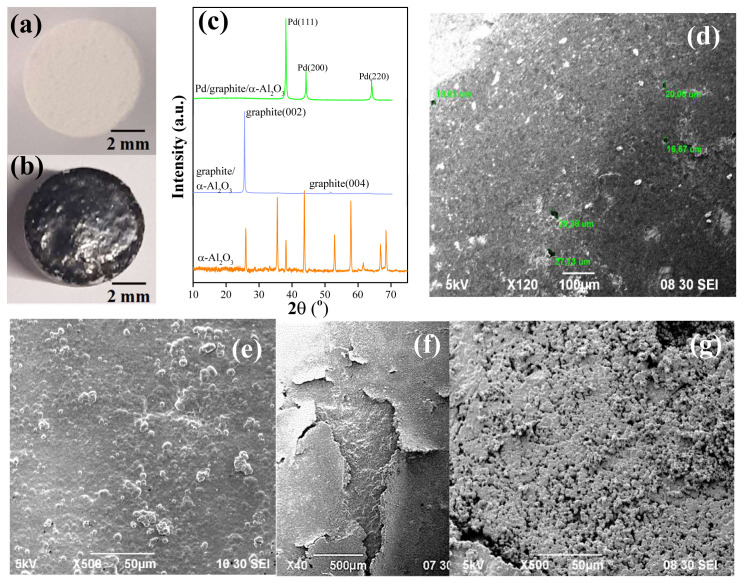
Comparison of the surfaces obtained using different techniques. Pictures of the tablet surface (a) before and (b) after graphite application, and (c) related XRD patterns; SEM images of the (d) graphite layer, Pd plated surface of (e) graphite covered sample (sample 14), (f) sample 14 zoomed out showing delamination, and (g) sample 10 after annealing.

To investigate the optimality of the new procedures developed in this study, the surface structure of the optimum sample (sample 13) was compared to the surfaces obtained (i) after annealing sample 10 at 700 °C for 24 h, and (ii) using the optimized ELP plating on a graphite interlayer (sample 14). Sample 10 was prepared using the ELP procedure described in the literature over a γ-Al_2_O_3_ interlayer, representing a comparison of conventional and optimized ELP methods. Sample 14, on the other hand, demonstrates the effectiveness of using γ-Al_2_O_3_ instead of other interlayer materials. The graphite layer in sample 14 was prepared by using a 2B pencil to draw on the α-Al_2_O_3_ surface, followed by calcination at 450 °C for 2 h to remove all organic impurities. The pictures and XRD patterns of the surface before and after graphite layer application can be seen in Figure 11, along with the SEM images of the graphite surface of sample 14 and the final Pd surfaces on samples 10 and 14.

Graphite application resulted in a thicker and denser intermediate layer compared to γ-Al_2_O_3_ application, as shown in Figure 11b. This can also be observed in the XRD patterns given in Figure 11c where no peaks from α-Al_2_O_3_ support can be observed after graphite application. The SEM image of this layer (Figure 11d) also supports the claim that a dense and homogeneous graphite interlayer was formed. Figure 11e shows that a smooth, dense and homogeneous Pd surface can be achieved by applying our optimized ELP procedure. On the other hand, annealing sample 10, which was prepared with the conventional ELP procedure, did not yield the expected surface structure. Comparing the surface states before (Figure 8a) and after (Figure 11g) annealing, it is seen that annealing resulted in a considerable improvement. However, the final surface is still significantly worse compared to the surfaces of samples 13 and 14.

The fact that a dense and homogeneous Pd surface was obtained on both γ-Al_2_O_3_ and graphite interlayers suggests that the optimized ELP procedure can be successfully applied on any surface. Nevertheless, it was observed that the graphite layer is loosely attached to the α-Al_2_O_3_ support, leading to delamination of the Pd surface easily (Figure 11f). This must be avoided to be able to use these structures as membranes. The average thickness of the Pd layer on these samples was calculated by the gravimetric method as described before. The calculated values were 26 μm, 13 μm, and 7 μm for Pd layer on α-Al_2_O_3_ (sample 10), γ-Al_2_O_3_/α-Al_2_O_3_ (sample 13), and graphite/α-Al_2_O_3_ (sample 14), respectively. It can be concluded that as the surface becomes smoother with smaller holes/pores/defects, a dense, uniform, and thinner Pd layer can be achieved.

## 4. Conclusions

Dense metallic membranes offer an alternative way to obtain pure H_2_ from mixture streams without any need to decrease the high pressure or temperature of these streams. However, the fact that Pd, which provides near-infinite permselectivity and high permeability for H_2_, is expensive has driven researchers to develop new membrane structures and preparation methods to achieve thinner Pd layers. To obtain such thin Pd layers, α-Al_2_O_3_ is widely studied as the support material due to its low cost and moldable structure. However, the large pores on the α-Al_2_O_3_ surface complicate the Pd plating process, resulting in nonselective transport across the membrane. This study investigated the methods to obtain a thin, dense, and homogeneous Pd layer by optimizing the support structure and ELP procedure.

To the best of our knowledge, this study provides the first detailed optimization study on obtaining dense Pd/γ-Al_2_O_3_/α-Al_2_O_3_ membrane structure using low-temperature plating, and investigating both the γ-Al_2_O_3_ layer formation and the ELP procedure. It has been concluded that PEG/PVA concentration (6-to-3 vol. %) and the slow heat treatment (1 °C/min, halting at 300 °C) are two important factors in achieving a thin and homogeneous γ-Al_2_O_3_ surface with smaller pores. The conventional ELP procedure was observed to have an extremely fast rate of reaction compared to that of mass transfer, resulting in bath decomposition and bulk Pd precipitation. To overcome this issue, the rate of reaction was slowed down to enhance mass transfer. This was achieved by decreasing the concentration of hydrazine from 3 M to 1 M, and sequentially introducing it to the plating bath. Additionally, the bath temperature was decreased from the widely used 50 °C to 30 °C. Furthermore, different from studies in the literature, the surface was equilibrated with a brief heat treatment at 120 °C between fresh plating baths, enabling the formation of the dense and uniform Pd layer (13-μm-thick) in three ELP baths without the need for annealing the sample for extended periods at elevated temperatures.

## Figures and Tables

**Table 1:** Contents and compositions of the solutions.

Solutions	Chemicals	Amount
Activating	Palladium (II) acetate	3.3 g
	Chloroform	100 mL

H_2_O_2_ solution	35% H_2_O_2_	3 g
	Deionized H_2_O	100 mL

Reducing	Deionized H_2_O	650 mL/L
	25% NH_4_OH	360 mL/L
	3 M hydrazine	10 mL/L

Plating	Deionized H_2_O	602 mL/L
	25% NH_4_OH	392 mL/L
	37% HCl	6 mL/L
	PdCl_2_	5.5 g/L
	3M hydrazine	1:100 ratio

**Table 2:** Summary of optimization parameters (all samples were dried at room temperature prior to calcination).

Sample no	PVA/PEG (vol./vol.) %	Application time (seconds)	Calcination
1	2 / 1	10	Direct method: heating rate 3 °C/min 3 h at 600 °C
2		20	
3		30	
4		60	

5	4 / 2	30	Direct method
6	4 / 2	30	Staged method: heating rate 1 °C/min i. 0.5 h at 300 °C ii. 3 h at 600 °C iii. 0.5 h at 300 °C iv. room temperature

7	6 / 3	30	Staged method
8	6 / 3	30 × 2	Staged method
9	8 / 4	30	Staged method

**Table 3:** Summary of optimization of ELP parameters and the obtained Pd crystallite sizes.

Sample no.	γ-Al_2_O_3_ layer	ELP Baths applied	Hydrazine concentration	Interstage heating step	Bath temperature (°C)	Crystallite size* (nm)
8	No	7	1 (vol.) % 3 M at once	No	50	-
9	No	7 (3 + 4)	1 (vol.) % 3 M at once	Between 3rd and 4th baths only	50	-

10	Yes	3	1 (vol.) % 3 M at once	Between every bath	50	-
11	Yes	3	1 (vol.) % 1 M, 0.99 (vol.) % 1 M, 0.98 (vol.) % 1 M	Between every bath	50	22

12	Yes	3	1 M, 1 M, 1 M	Between every bath	40	15
13	Yes	3	1 M, 1 M, 1 M	Between every bath	30	16

* Calculated using Scherrer equation with significant XRD peaks at 2θ @ 40°, 47°.

## References

[1] Al Mufachi NA, Rees NV, Steinberger Wilkens R (2015). Hydrogen selective membranes: A review of palladium-based dense metal membranes. Renewable and Sustainable Energy Reviews.

[2] Guo Y, Wu H, Fan X, Zhou L, Chen Q (2017). Palladium composite membrane fabricated on rough porous alumina tube without intermediate layer for hydrogen separation. International Journal of Hydrogen Energy.

[3] Momirlan M, Veziroglu TN (2002). Current status of hydrogen energy. Renewable and Sustainable Energy Reviews.

[4] Edwards PP, Kuznetsov VL, David WIF, Brandon NP (2008). Hydrogen and fuel cells: Towards a sustainable energy future. Energy Policy.

[5] Dincer I, Rosen MA (2011). Sustainability aspects of hydrogen and fuel cell systems. Energy for Sustainable Development.

[6] Rahimpour MR, Samimi F, Babapoor A, Tohidian T, Mohebi S (2017). Palladium membranes applications in reaction systems for hydrogen separation and purification: A review. Chemical Engineering and Processing: Process Intensification.

[7] Ockwig NW, Nenoff TM (2007). Membranes for Hydrogen Separation. Chemical Reviews.

[8] Li P, Wang Z, Qiao Z, Liu Y, Cao X (2015). Recent developments in membranes for efficient hydrogen purification. Journal of Membrane Science.

[9] Conde JJ, Maroño M, Sánchez Hervás JM (2017). Pd-Based Membranes for Hydrogen Separation: Review of Alloying Elements and Their Influence on Membrane Properties. Separation & Purification Reviews.

[10] Paglieri SN, Way JD (2002). Innovations in palladium membrane research. Separation and Purification Methods.

[11] Okazaki J, Ikeda T, Tanaka DAP, Sato K, Suzuki TM (2011). An investigation of thermal stability of thin palladium–silver alloy membranes for high temperature hydrogen separation. Journal of Membrane Science.

[12] Lee G, Easa J, Jin R, Booth A, O’Brien CP (2021). Enhancing the surface sensitivity of in-situ/operando characterization of palladium membranes through polarization modulation and synthesis of optically smooth palladium thin films. Journal of Membrane Science.

[13] Yun S, Ted Oyama S (2011). Correlations in palladium membranes for hydrogen separation: A review. Journal of Membrane Science.

[14] Li A, Liang W, Hughes R (2000). Fabrication of dense palladium composite membranes for hydrogen separation. Catalysis Today.

[15] Lim H, Gu Y, Oyama ST (2012). Studies of the effect of pressure and hydrogen permeance on the ethanol steam reforming reaction with palladium- and silica-based membranes. Journal of Membrane Science.

[16] Paglieri SN, Foo KY, Way JD, Collins JP, Harper Nixon DL (1999). A New Preparation Technique for Pd/Alumina Membranes with Enhanced High-Temperature Stability. Industrial & Engineering Chemistry Research.

[17] Kitiwan M, Atong D (2010). Effects of Porous Alumina Support and Plating Time on Electroless Plating of Palladium Membrane. Journal of Materials Science & Technology.

[18] Collins JP, Way JD (1993). Preparation and characterization of a composite palladium-ceramic membrane. Industrial & Engineering Chemistry Research.

[19] Shi Z, Wu S, Szpunar JA, Roshd M (2006). An observation of palladium membrane formation on a porous stainless steel substrate by electroless deposition. Journal of Membrane Science.

[20] Pal N, Agarwal M, Maheshwari K, Solanki YS (2020). A review on types, fabrication and support material of hydrogen separation membrane. Materials Today: Proceedings.

[21] Pan XL, Xiong GX, Sheng SS, Stroh N, Brunner H (2001). Thin dense Pd membranes supported on α-Al2O3 hollow fibers. Chemical Communications.

[22] Gu Y, Ted Oyama S (2007). Ultrathin, hydrogen-selective silica membranes deposited on alumina-graded structures prepared from size-controlled boehmite sols. Journal of Membrane Science.

[23] Khanmohammadi S, Taheri Nassaj E, Farrokhi Rad M (2020). Synthesis of meso-porous gamma-alumina membrane: effect of yttria addition on the thermal stability. Surfaces and Interfaces.

[24] Zhao HB, Pflanz K, Gu JH, Li AW, Stroh N (1998). Preparation of palladium composite membranes by modified electroless plating procedure. Journal of Membrane Science.

[25] Zhang X, Xiong G, Yang W (2008). A modified electroless plating technique for thin dense palladium composite membranes with enhanced stability. Journal of Membrane Science.

[26] Pacheco Tanaka DA, Llosa Tanco MA, Okazaki J, Wakui Y, Mizukami F (2008). Preparation of “pore-fill” type Pd–YSZ–γ-Al2O3 composite membrane supported on α-Al2O3 tube for hydrogen separation. Journal of Membrane Science.

[27] Nair BKR, Choi J, Harold MP (2007). Electroless plating and permeation features of Pd and Pd/Ag hollow fiber composite membranes. Journal of Membrane Science.

[28] Lim H, Oyama ST (2011). Hydrogen selective thin palladium–copper composite membranes on alumina supports. Journal of Membrane Science.

[29] Pacheco Tanaka DA, Medrano JA, Viviente Sole JL, Gallucci F, Basile A, Gallucci F (2020). 1Metallic membranes for hydrogen separation. Current Trends and Future Developments on (Bio-) Membranes.

[30] Abu El Hawa HW, Lundin STB, Paglieri SN, Harale A, Douglas Way J (2015). The influence of heat treatment on the thermal stability of Pd composite membranes. Journal of Membrane Science.

[31] Yeung KL, Christiansen SC, Varma A (1999). Palladium composite membranes by electroless plating technique: Relationships between plating kinetics, film microstructure and membrane performance. Journal of Membrane Science.

[32] Guazzone F, Ma YH (2008). Leak growth mechanism in composite Pd membranes prepared by the electroless deposition method. AIChE Journal.

[33] Mardilovich PP, She Y, Ma YH, Rei MH (1998). Defect-free palladium membranes on porous stainless-steel support. AIChE Journal.

[34] Pacheco Tanaka DA, Llosa Tanco MA, Niwa SI, Wakui Y, Mizukami F (2005). Preparation of palladium and silver alloy membrane on a porous α-alumina tube via simultaneous electroless plating. Journal of Membrane Science.

[35] Volpe M, Inguanta R, Piazza S, Sunseri C (2006). Optimized bath for electroless deposition of palladium on amorphous alumina membranes. Surface and Coatings Technology.

[36] Gade SK, Thoen PM, Way JD (2008). Unsupported palladium alloy foil membranes fabricated by electroless plating. Journal of Membrane Science.

[37] Thoen PM, Roa F, Way JD (2006). High flux palladium–copper composite membranes for hydrogen separations. Desalination.

[38] Rhoda RN (1959). Electroless Palladium Plating. Transactions of the IMF.

[39] Keuler J, Lorenzen L, Sanderson R, Linkov V (1997). Optimizing palladium conversion in electroless palladium plating of alumina membranes. Plating and surface finishing.

[40] Yong CC, Wang J (2001). Mechanical-Activation-Triggered Gibbsite-to-Boehmite Transition and Activation-Derived Alumina Powders. Journal of the American Ceramic Society.

[41] Brostow W, Datashvili T (2008). Chemical modification and characterization of boehmite particles. Chemistry and Chemical Technology.

[42] He Z, Ng TCA, Lyu Z, Gu Q, Zhang L (2020). Alumina double-layered ultrafiltration membranes with enhanced water flux. Colloids and Surfaces A: Physicochemical and Engineering Aspects.

[43] Zanganeh S, Kajbafvala A, Zanganeh N, Mohajerani MS, Lak A (2010). Self-assembly of boehmite nanopetals to form 3D high surface area nanoarchitectures. Applied Physics A.

[44] Omori T, Kusama T, Kawata S, Ohnuma I, Sutou Y (2013). Abnormal Grain Growth Induced by Cyclic Heat Treatment. Science.

[45] Eka Perkasa D, Qonita A, Soegijono B (2021). Influence Of Heat Treatment On Crystal Structure And Corrosion Properties Of Co-Cr-Mo-Al Alloy In Simulated Body Fluid Solution. Journal of Physics: Conference Series.

[46] Ryi SK, Xu N, Li A, Lim CJ, Grace JR (2010). Electroless Pd membrane deposition on alumina modified porous Hastelloy substrate with EDTA-free bath. International Journal of Hydrogen Energy.

[47] Wang JY, Chi YH, Huang JH (2021). Electroless Plating of High-Performance Composite Pd Membranes with EDTA-Free Bath. Materials.

[48] McCool BA, Lin YS (2001). Nanostructured thin palladium-silver membranes: Effects of grain size on gas permeation properties. Journal of Materials Science.

[49] Murthy Sanjeeva, Jaffe M, Hammond W, Tolias P, Arinzeh T (2013). 2- Scattering techniques for structural analysis of biomaterials. Characterization of Biomaterials.

[50] Sahoo B, Joseph J, Sharma A, Paul J (2019). Particle size and shape effects on the surface mechanical properties of aluminium coated with carbonaceous materials. Journal of Composite Materials.

[51] Slama C, Jaafar H, Karouia A, Abdellaoui M (2021). Diffraction Crystallite Size Effects on Mechanical Properties of Nanocrystalline (Ti0.8W0.2)C. Chemistry Africa.

